# Supplementary material for: Spatial control of avidity regulates initiation and progression of selective autophagy
Source: Nat Commun. 2021 Dec 10;12:7194. doi: 10.1038/s41467-021-27420-3 (PMC8664900; doi:10.1038/s41467-021-27420-3)
Supplement: Supplementary file 1 — Supplementary Information [file 41467_2021_27420_MOESM1_ESM.pdf]

Supplementary Information to:

Spatial control of avidity regulates initiation and progression of selective autophagy

David M. Hollenstein[1,2,3*], Mariya Licheva[3,4,*], Nicole Konradi[3], David Schweida[1], Hector Mancilla[3], Muriel Mari[5], Fulvio Reggiori[5], and Claudine Kraft[3,6,7,§]

Index

Supplementary Fig. 1 (Data related to Fig. 1): Atg11-µNS forms large cytosolic particles that retain the characteristic protein interactions of Atg11. 2

Supplementary Fig. 2 (Data related to Fig. 2): Vac8 is required for the vacuole recruitment of the selective PAS. 4

Supplementary Fig. 3 (Data related to Fig. 2): Vac8 anchors the selective PAS by interacting with the N-terminus of Atg11. 6

Supplementary Fig. 4 (Data related to Fig. 3): The interaction of Atg11 with Vac8 is direct and of low affinity....8

Supplementary Fig. 5 (Data related to Fig. 4): Loss of Vac8 prevents PAS and vacuole recruitment of the PI3K complex. 10

Supplementary Fig. 6 (Data related to Fig. 5): Vac8 recruits the PI3K complex independently of autophagy. 12

Supplementary Fig. 7 (Data related to Fig. 5): Artificial tethering of Atg11 or Atg14 restores vacuole localization in the absence of Vac8. 14

Supplementary Fig. 8 (Data related to Fig. 6): Reconstitution of PAS formation at the nuclear membrane is sufficient for PAS assembly. 16

Supplementary Fig. 9 (Data related to Fig. 6): Reconstitution of PAS formation at the nuclear membrane is sufficient for autophagosome formation. 18

Supplementary Fig. 1

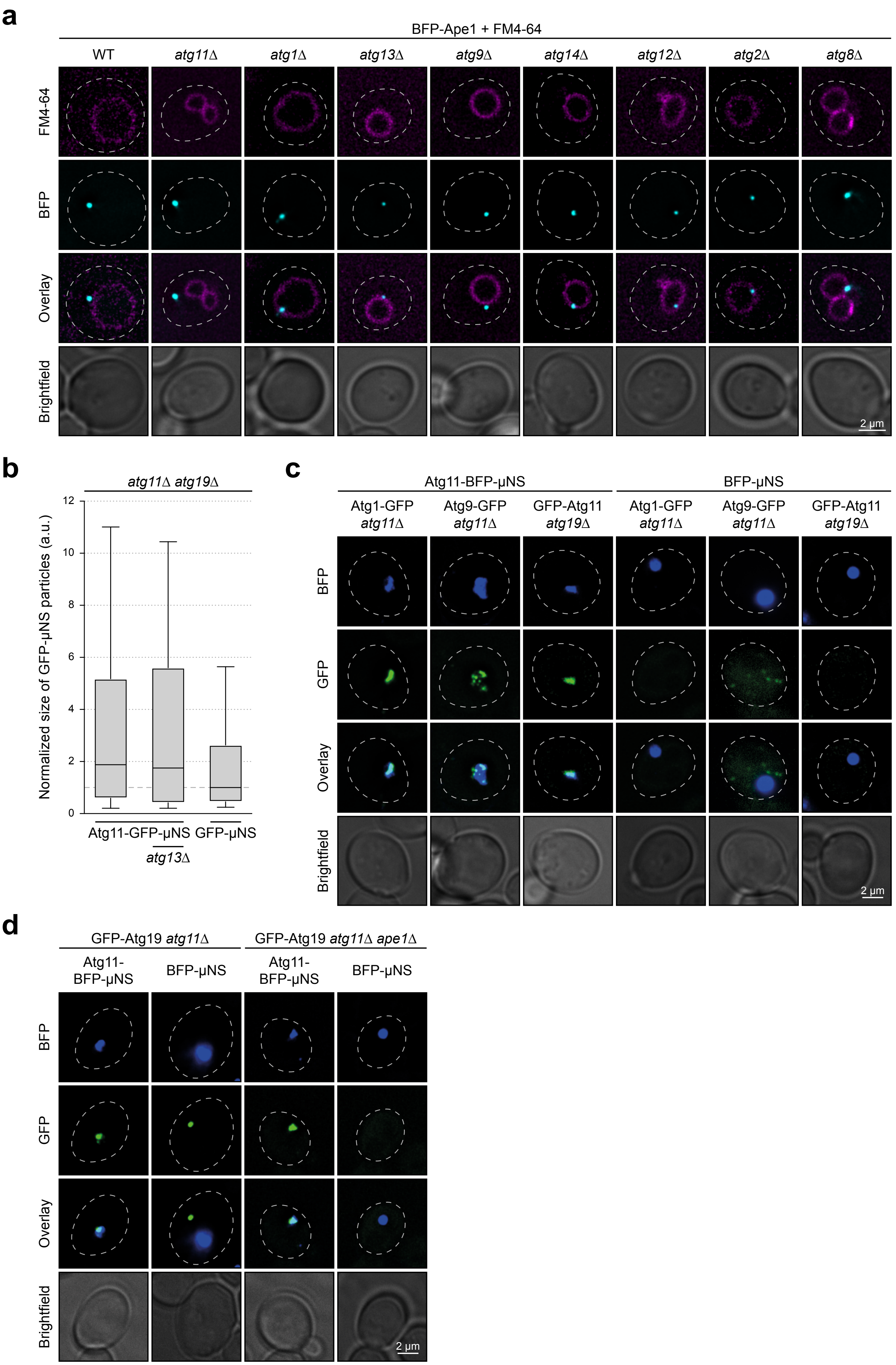

Supplementary Fig. 1 (Data related to Fig. 1): Atg11-µNS forms large cytosolic particles that retain the characteristic protein interactions of Atg11.

a Individual fluorescence microscopy channels and brightfield images of cells shown in Fig. 1a. Dashed lines indicate the contour of individual cells.

b Boxplots of µNS particle size. At least 500 µNS particles from three independent biological replicates were analysed per strain. All values were normalized to the median size of GFP-µNS particles. Dark horizontal lines represent medians, boxes represent the 25th and 75th percentiles, whiskers expand to the 10th and 90th percentiles; outliers are not shown. a.u., arbitrary units. See Fig. 1d for representative fluorescence microscopy images.

c Atg1-GFP *atg11Δ*, Atg9-GFP *atg11Δ* and GFP-Atg11 *atg19Δ* cells containing either Atg11-BFP-µNS or BFP-µNS were grown to mid log-phase. Representative fluorescence microscopy images of one out of three independent experiments are shown. Dashed lines indicate the contour of individual cells.

d GFP-Atg19 *atg11Δ* or GFP-Atg19 *atg11Δ ape1Δ* cells containing either Atg11-BFP-µNS or BFP-µNS were grown to mid log-phase. Representative fluorescence microscopy images of one out of three independent experiments are shown. Dashed lines indicate the contour of individual cells.

Supplementary Fig. 2

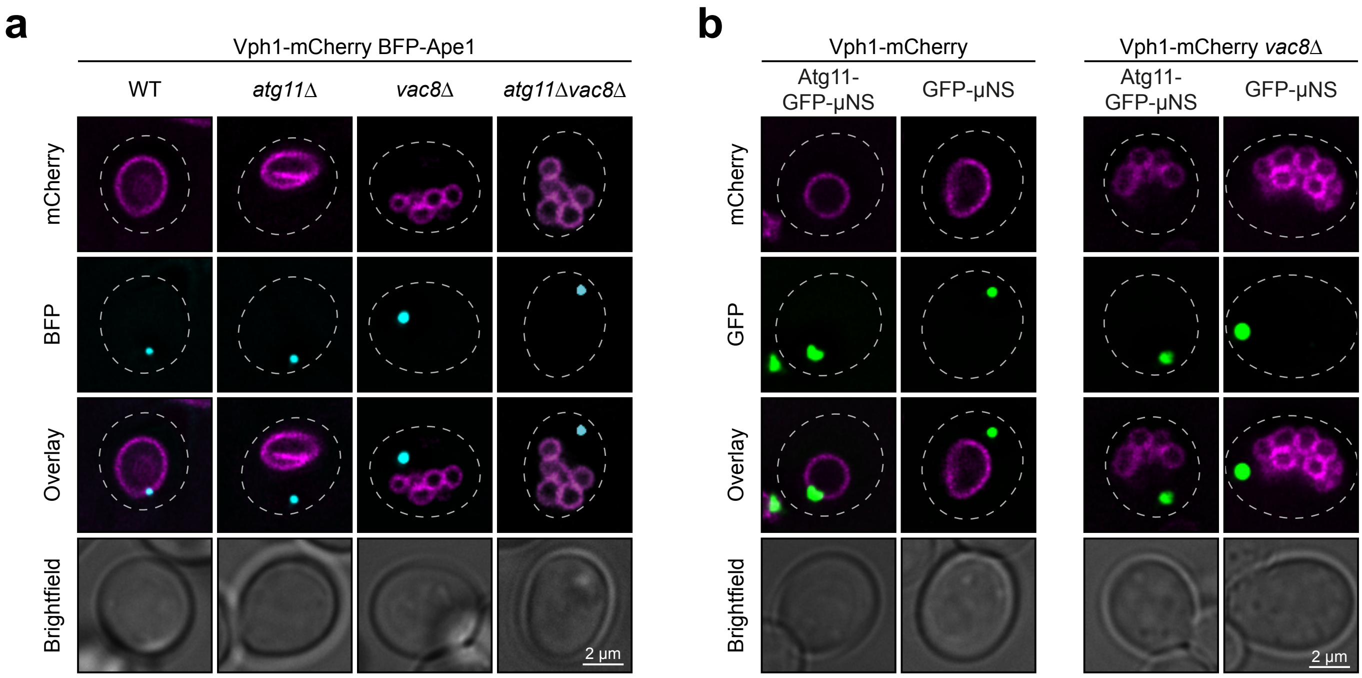

Supplementary Fig. 2 (Data related to Fig. 2): Vac8 is required for the vacuole recruitment of the selective PAS.

a Individual fluorescence microscopy channels and brightfield images of cells shown in Fig. 2a and 2b. Representative fluorescence microscopy images of one out of three independent experiments are shown. Dashed lines indicate the contour of individual cells.
b Vph1-4xmCherry or Vph1-4xmCherry *vac8Δ* cells containing Atg11-GFP-µNS or GFP-µNS were grown to mid-log phase. The intracellular localization of µNS particles was analysed by fluorescence microscopy. Representative fluorescence microscopy images of one out of three independent experiments are shown. Dashed lines indicate the contour of individual cells.

Supplementary Fig. 3

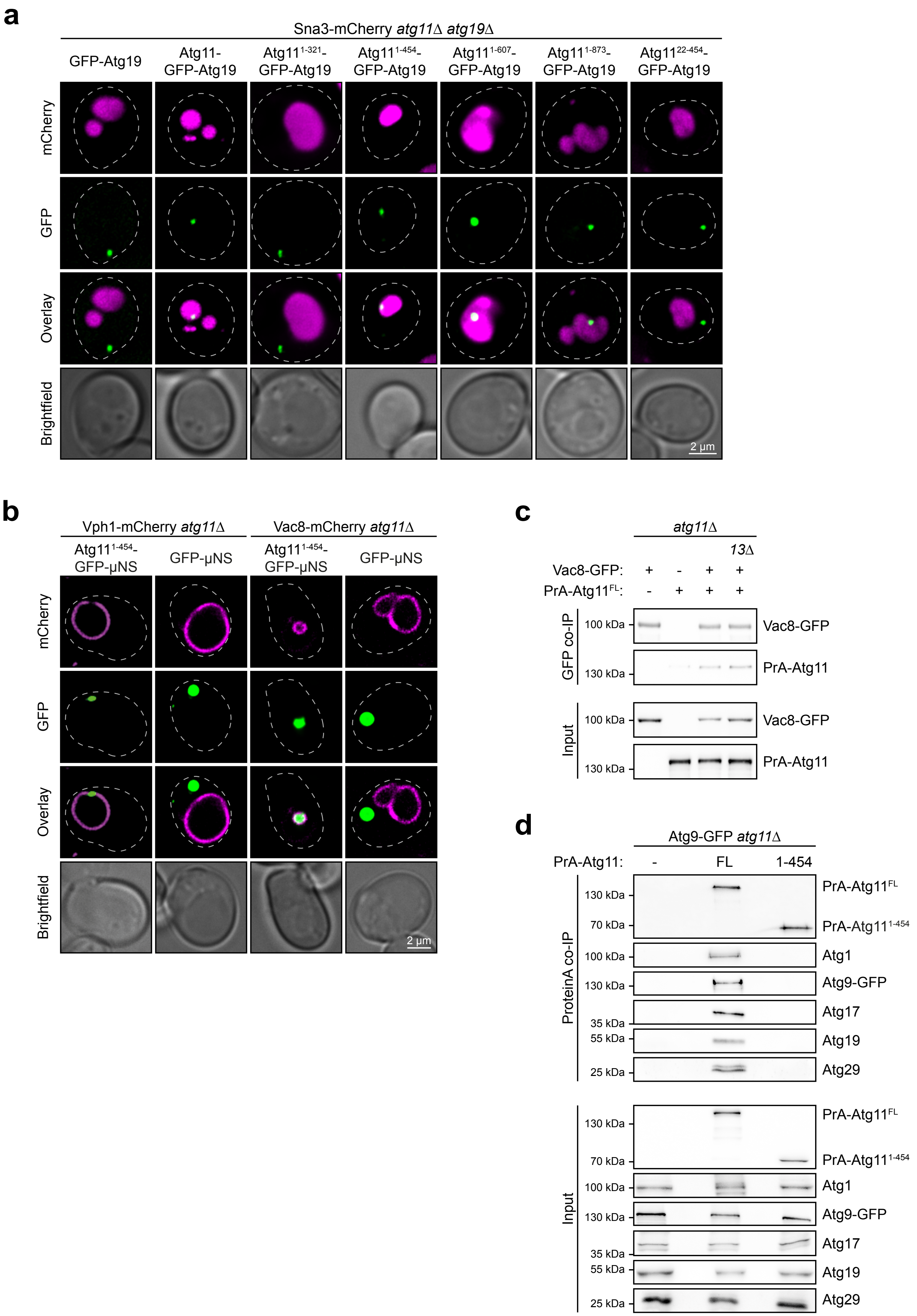

Supplementary Fig. 3 (Data related to Fig. 2): Vac8 anchors the selective PAS by interacting with the N-terminus of Atg11.

a Representative fluorescence microscopy images of the quantification in Fig. 2f are shown. Dashed lines indicate the contour of individual cells.
b Vph1-4xmCherry *atg11Δ* or Vac8-mCherry *atg11Δ* cells containing Atg11$^{1-454}$-GFP-µNS or GFP-µNS were grown to mid-log phase. The intracellular localization of µNS particles and the vacuolar distribution of Vph1-4xmCherry or Vac8-mCherry was analysed by fluorescence microscopy. Representative fluorescence microscopy images of one out of three independent experiments are shown. Dashed lines indicate the contour of individual cells.
c Vac8-GFP *atg11Δ,* Vac8-GFP *atg11Δ atg13Δ* or *atg11Δ* cells containing 2xProteinA-Atg11^{FL} or an empty plasmid were grown to mid-log phase, followed by glass bead lysis. Vac8-GFP was immunoprecipitated and the amount of precipitated Vac8-GFP and co-precipitated 2xProteinA-Atg11^{FL} was analysed by anti-GFP and anti-protein A western blotting. One representative experiment out of two independent experiments is shown. FL, full length; PrA, protein A.
d Atg9-GFP *atg11Δ* cells containing an empty plasmid, 2xProteinA-Atg11^{FL} or 2xProteinA-Atg11$^{1-454}$ were grown to mid-log phase. Cell extracts were prepared by cryo-milling followed by Protein A immunoprecipitation. The amount of precipitated protein was measured by anti-protein A western blotting, and the co-precipitation of known Atg11 interaction partners was analysed. One representative experiment out of three independent experiments is shown. FL, full length; PrA, protein A.

Supplementary Fig. 4

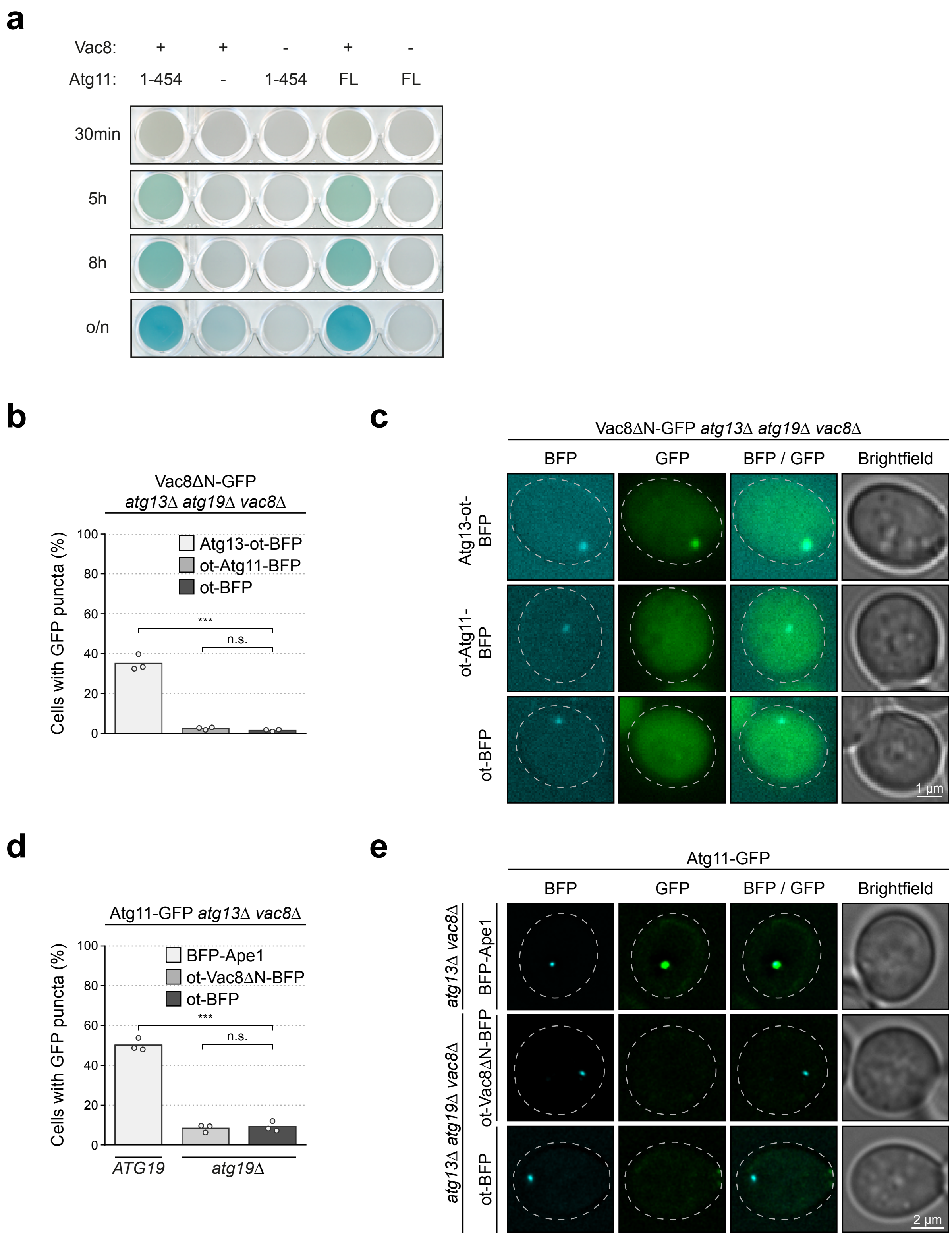

Supplementary Fig. 4 (Data related to Fig. 3): The interaction of Atg11 with Vac8 is direct and of low affinity.

a The binding of Vac8 to $Atg11^{FL}$ or to $Atg11^{1-454}$ was examined by yeast two-hybrid analysis. The interaction was assessed by analysing β-galactosidase activity (blue signal). One out of three representative experiments is shown. FL, full length

b and **c** *atg13Δ atg19Δ vac8Δ* cells containing Vac8ΔN-GFP and Atg13-ot-mTagBFP2 (Atg13-$Atg19^{152-191}$-mTagBFP2), ot-Atg11-mTagBFP2 or ot-mTagBFP2 were grown to mid-log phase. The recruitment of Vac8ΔN-GFP to BFP puncta was analysed. **b** The percentage of cells with GFP puncta was analysed in three independent biological replicates. For each condition and replicate at least 100 cells were analyzed. The values of each replicate (circle) and the mean (bars) were plotted. Statistical analysis using two-tailed unpaired t-tests. Significance is indicated with asterisks: *** $p < 0.001$, ** $p < 0.01$, * $p < 0.05$, n.s. (not significant) $p > 0.05$. Exact numerical values are reported in the source data. **c** Representative fluorescence microscopy images are shown. Dashed lines indicate the contour of individual cells. ot, oligomer tether.

d and **e** *atg13Δ atg19Δ vac8Δ* cells containing Atg11-GFP and ot-Vac8ΔN-BFP or ot-BFP, and *atg13Δ vac8Δ* cells expressing mTagBFP2-Ape1 and Atg11-GFP were grown to mid-log phase. The recruitment of Atg11-GFP to BFP puncta was analysed. **d** The percentage of cells with GFP puncta was analysed in three independent biological replicates. For each condition and replicate at least 100 cells were analyzed. The values of each replicate (circle) and the mean (bars) were plotted. Statistical analysis using two-tailed unpaired t-tests. Significance is indicated with asterisks: *** $p < 0.001$, ** $p < 0.01$, * $p < 0.05$, n.s. (not significant) $p > 0.05$. Exact numerical values are reported in the source data. **e** Representative fluorescence microscopy images are shown. Dashed lines indicate the contour of individual cells. ot, oligomer tether.

Supplementary Fig. 5

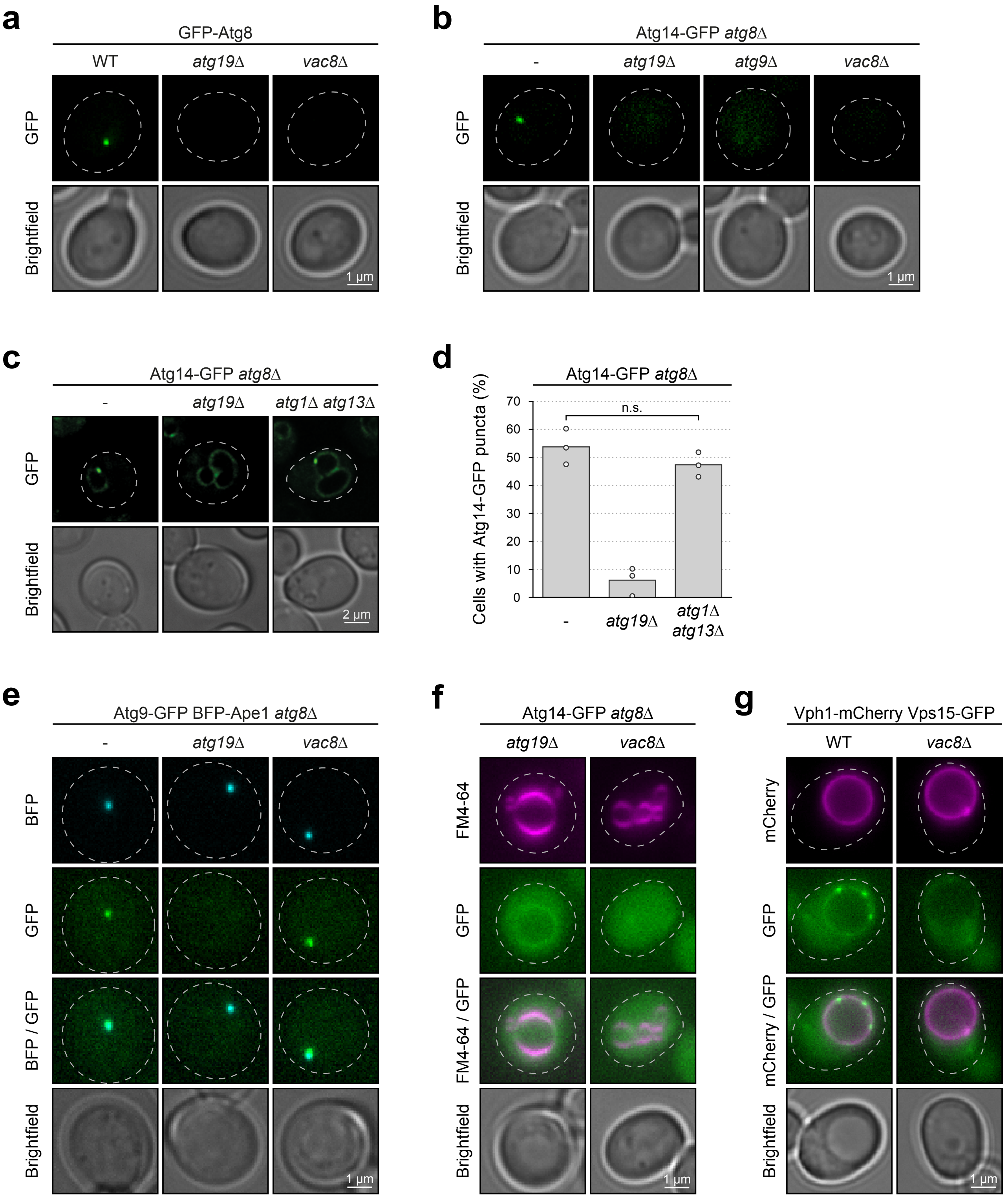

Supplementary Fig. 5 (Data related to Fig. 4): Loss of Vac8 prevents PAS and vacuole recruitment of the PI3K complex.

a Representative fluorescence microscopy images of the quantification in Fig. 4b are shown. Dashed lines indicate the contour of individual cells.
b Representative fluorescence microscopy images of the quantification in Fig. 4c are shown. Dashed lines indicate the contour of individual cells.
c and **d** *atg8Δ, atg8Δ atg19Δ* and *atg1Δ atg8Δ atg13Δ* cells containing Atg14-3xGFP were grown to mid log-phase. **c** Representative fluorescence microscopy images are shown. Dashed lines indicate the contour of individual cells. **d** The percentage of cells with GFP puncta was analysed in three independent biological replicates. For each condition and replicate at least 200 cells were analyzed. The values of each replicate (circle) and the mean (bars) were plotted. Statistical analysis using two-tailed unpaired t-tests. Significance is indicated with asterisks: *** $p < 0.001$, ** $p < 0.01$, * $p < 0.05$, n.s. (not significant) $p > 0.05$. Exact numerical values are reported in the source data.
e Representative fluorescence microscopy images of the quantification in Fig. 4d are shown. Dashed lines indicate the contour of individual cells.
f Individual fluorescence microscopy channels and brightfield images of cells shown in Fig 4e. Dashed lines indicate the contour of individual cells. One representative experiment out of two independent experiments is shown.
g Individual fluorescence microscopy channels and brightfield images of cells shown in Fig 4g. Dashed lines indicate the contour of individual cells. One representative experiment out of two independent experiments is shown.

Supplementary Fig. 6

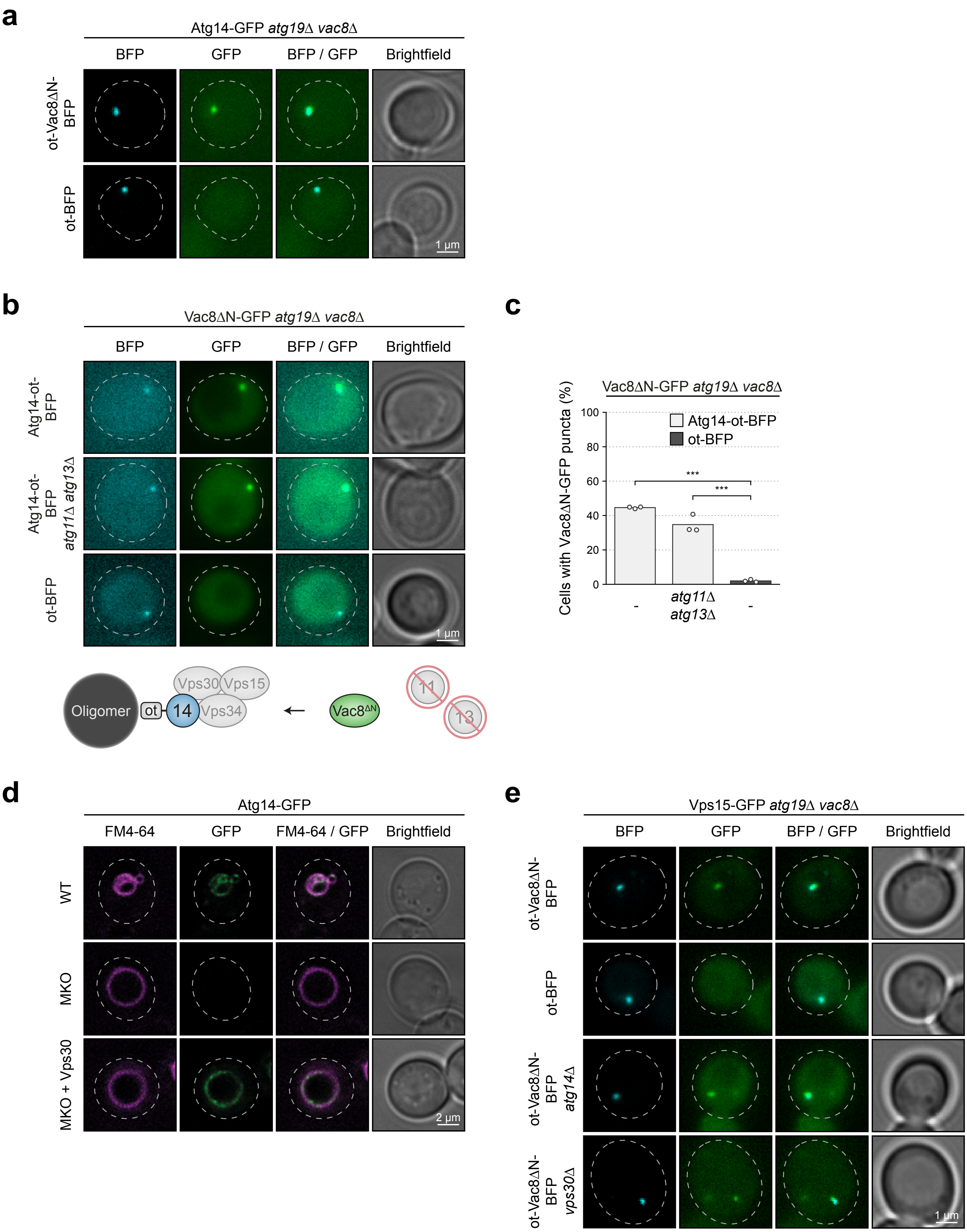

Supplementary Fig. 6 (Data related to Fig. 5): Vac8 recruits the PI3K complex independently of autophagy.

a Representative fluorescence microscopy images of the quantification in Fig. 5a are shown. Dashed lines indicate the contour of individual cells. ot, oligomer tether.

b and **c** The indicated strains containing Vac8ΔN-GFP and Atg14-ot-mTagBFP2 or ot-mTagBFP2 were grown to mid-log phase. The recruitment of Vac8ΔN-GFP to BFP puncta was analysed. **b** Representative fluorescence microscopy images are shown. Dashed lines indicate the contour of individual cells. **c** The percentage of cells with GFP puncta was analysed in three independent biological replicates. For each condition and replicate at least 100 cells were analyzed. The values of each replicate (circle) and the mean (bars) were plotted. Statistical analysis using two-tailed unpaired t-tests. Significance is indicated with asterisks: *** $p < 0.001$, ** $p < 0.01$, * $p < 0.05$, n.s. (not significant) $p > 0.05$. Exact numerical values are reported in the source data.

d Wild type cells containing Atg14-3xGFP and an empty plasmid or multiple knock-out (MKO) cells, containing either Atg14-3xGFP and an empty plasmid or Atg14-3xGFP and Vps30-2xProteinA, were grown to mid log-phase. Vacuoles were stained with FM4-64. Dashed lines indicate the contour of individual cells. Representative fluorescence microscopy images of one out of three independent experiments are shown. MKO: *atg1Δ 2Δ 3Δ 4Δ 5Δ 6Δ/vps30Δ 7Δ 8Δ 9Δ 10Δ 11Δ 12Δ 13Δ 14Δ 16Δ 17Δ 18Δ 19Δ 20Δ 21Δ 23Δ 24Δ 27Δ 29Δ 31Δ*::HIS3

e Representative fluorescence microscopy images of the quantification in Fig. 5b are shown. Dashed lines indicate the contour of individual cells.

Supplementary Fig. 7

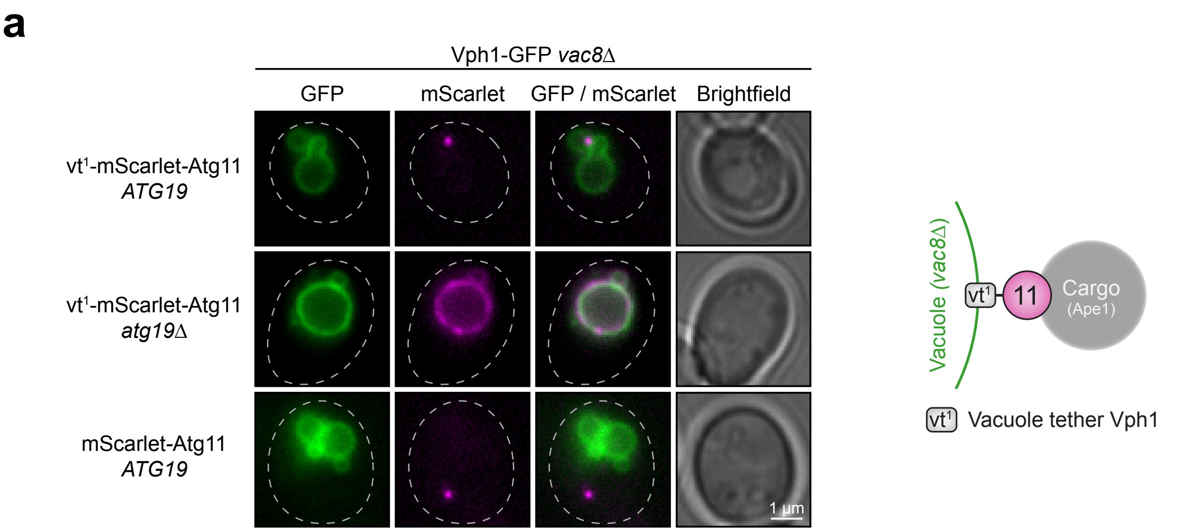

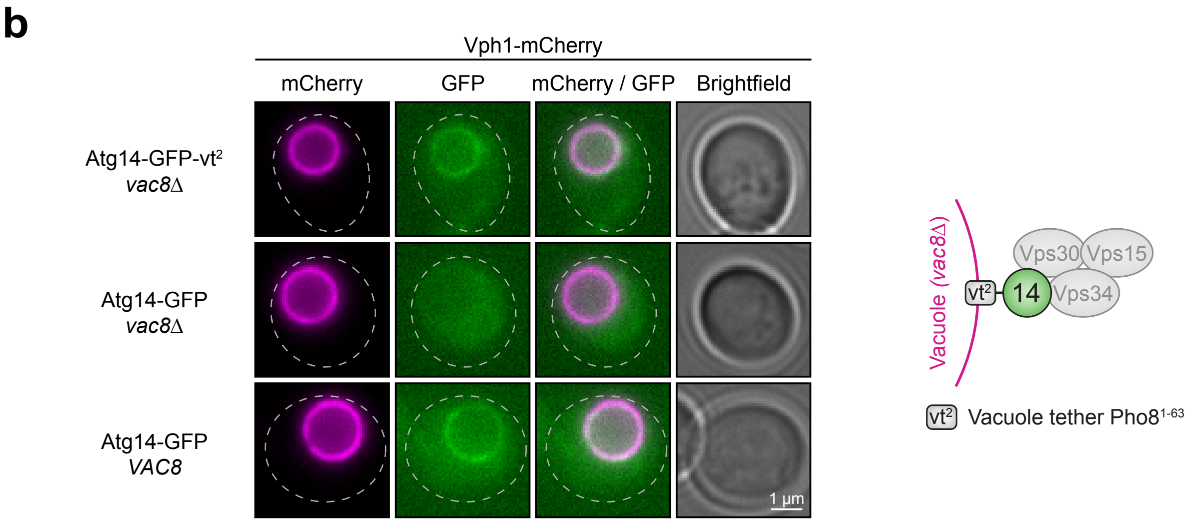

Supplementary Fig. 7 (Data related to Fig. 5): Artificial tethering of Atg11 or Atg14 restores vacuole localization in the absence of Vac8.

a The indicated Vph1-GFP strains containing Vph1-mScarlet-Atg11 or mScarlet-Atg11 were analysed. Representative microscopy images of one experiment out of two independent experiments are shown. Dashed lines indicate the contour of individual cells. A schematic of the experimental setup is shown on the right. vt^1, vacuole tether Vph1.
b The indicated Vph1-4xmCherry strains containing Atg14-GFP-$Pho8^{1\text{-}63}$ or Atg14-GFP were analysed. Representative microscopy images of one experiment out of two independent experiments are shown. Due to the low visibility of Atg14-GFP and Atg14-GFP-$Pho8^{1\text{-}63}$ in fluorescence microscopy, these constructs were expressed under the higher expression *VAC8* promoter. Dashed lines indicate the contour of individual cells. A schematic of the experimental setup is shown on the right. vt^2, vacuole tether $Pho8^{1\text{-}63}$.

Supplementary Fig. 8

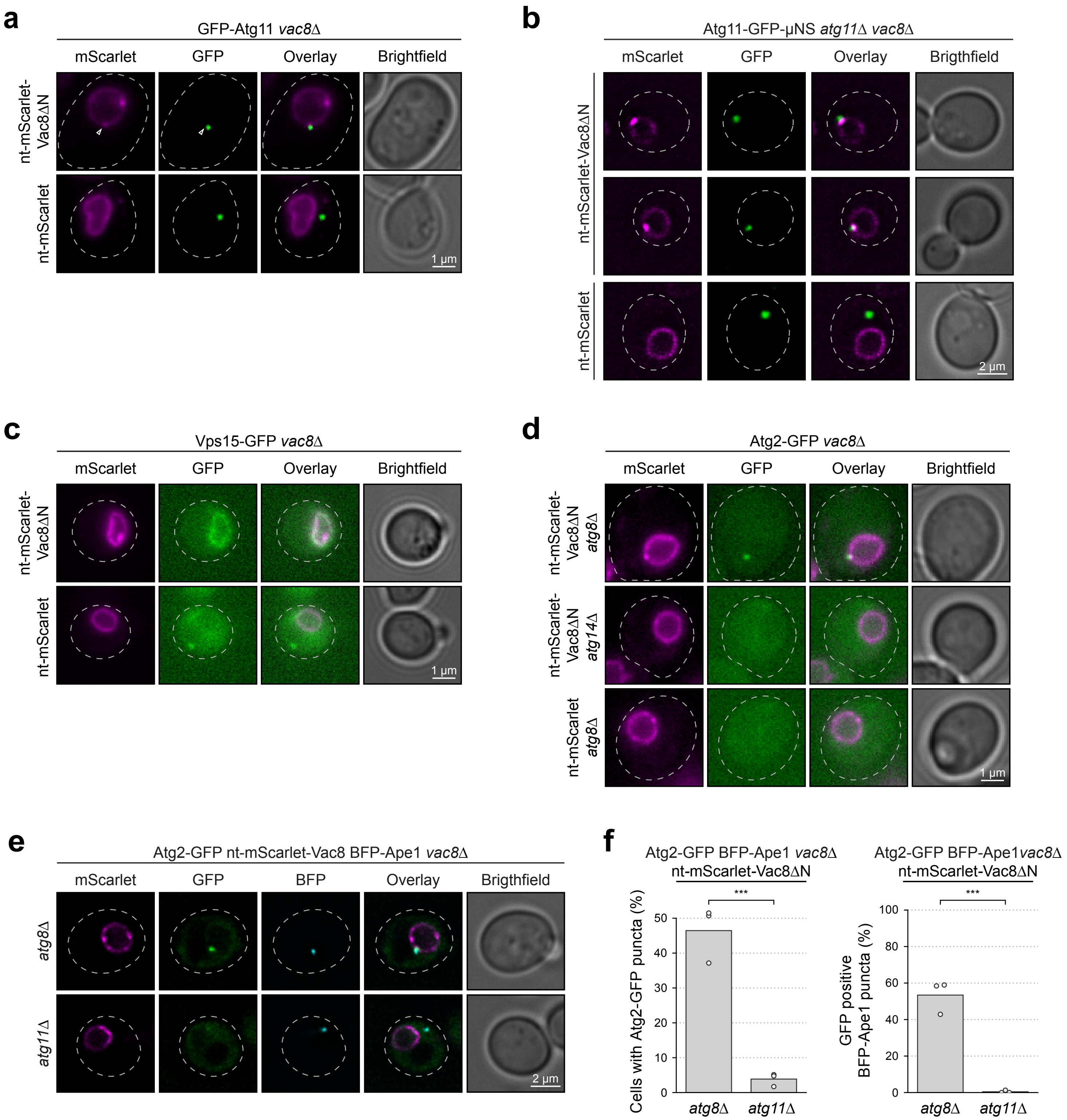

Supplementary Fig. 8 (Data related to Fig. 6): Reconstitution of PAS formation at the nuclear membrane is sufficient for PAS assembly.

a Individual fluorescence microscopy channels and brightfield images of the quantification in Fig 6b are shown. Dashed lines indicate the contour of individual cells, white arrows indicate the enrichment of nt-mScarlet-Vac8ΔN at the interaction site with a GFP-Atg11 punctum. nt, nucleus tether ($Nvj1^{1-125}$).
b *atg11*Δ *vac8*Δ cells containing Atg11-GFP-µNS and nt-mScarlet-Vac8ΔN or nt-mScarlet were grown to mid-log phase. The recruitment of Atg11-GFP-µNS particles to the nuclear membrane and the enrichment of nt-mScarlet-Vac8ΔN at the particle interaction site was analysed. Representative microscopy images of one out of two independent experiments are shown. Dashed lines indicate the contour of individual cells.
c Individual fluorescence microscopy channels and brightfield images of the quantification in Fig 6c are shown. Dashed lines indicate the contour of individual cells.
d Individual fluorescence microscopy channels and brightfield images of the quantification in Fig 6d are shown. Dashed lines indicate the contour of individual cells.
e and **f** Atg2-GFP mTagBFP2-Ape1 *atg8Δ vac8Δ* or Atg2-GFP mTagBFP2-Ape1 *atg11Δ vac8Δ* cells containing nt-mScarlet-Vac8ΔN were grown to mid log-phase. The formation of Atg2-GFP puncta and the overlap of mTagBFP2-Ape1 puncta with Atg2-GFP puncta was analyzed. **e** Representative fluorescence microscopy images are shown. Dashed lines indicate the contour of individual cells. **f** The percentage of cells with Atg2-GFP puncta (left) and the percentage of Atg2-GFP positive mTagBFP2-Ape1 puncta (right) was analysed in three independent biological replicates. For each condition and replicate at least 100 cells and 75 BFP puncta were analyzed. The values of each replicate (circle) and the mean (bars) were plotted. Statistical analysis using two-tailed unpaired t-tests. Significance is indicated with asterisks: *** $p < 0.001$, ** $p < 0.01$, * $p < 0.05$, n.s. (not significant) $p > 0.05$. Exact numerical values are reported in the source data.

Supplementary Fig. 9

a

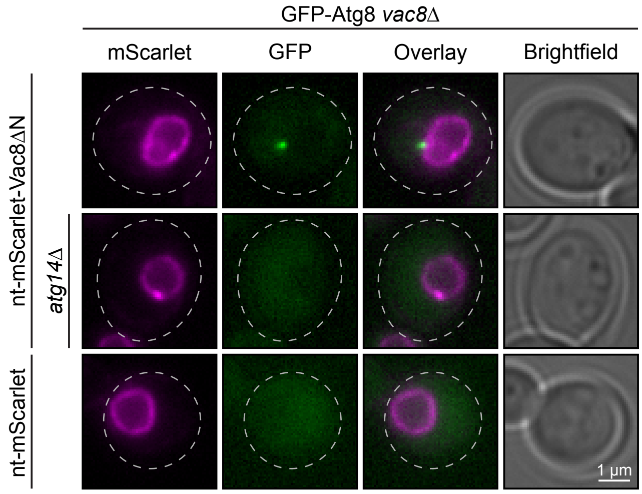

b

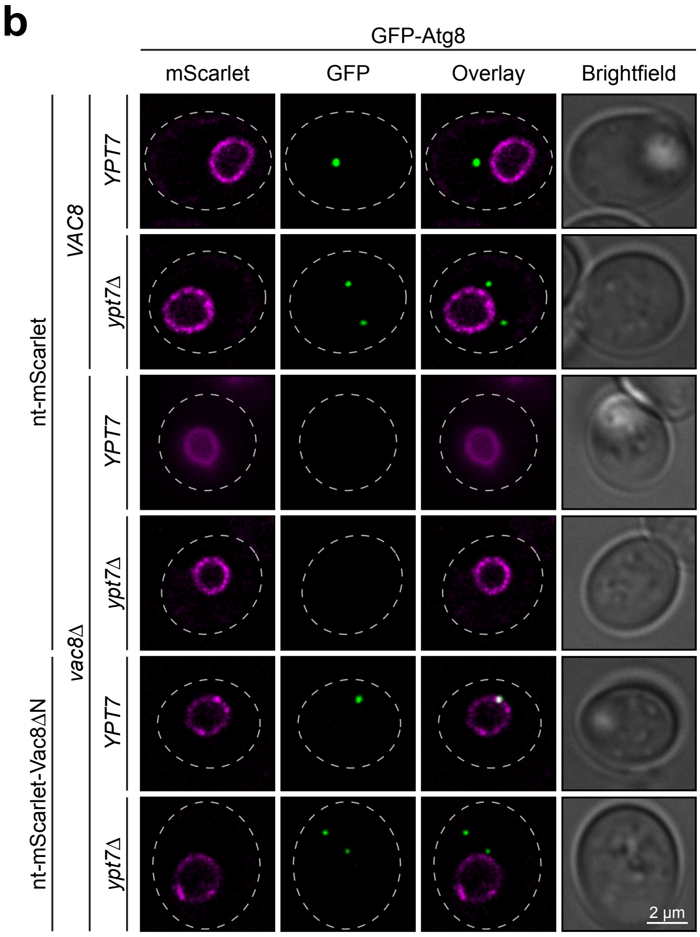

c

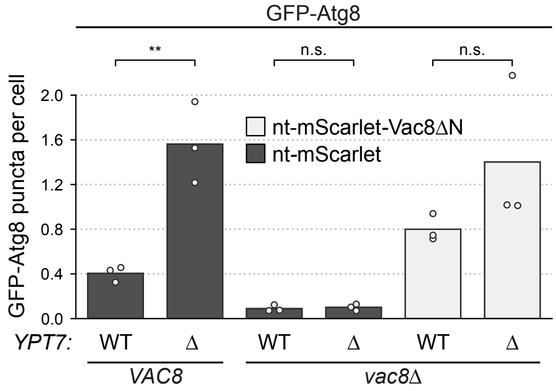

Supplementary Fig. 9 (Data related to Fig. 6): Reconstitution of PAS formation at the nuclear membrane is sufficient for autophagosome formation.

a Individual fluorescence microscopy channels and brightfield images of the quantification in Fig 6e are shown. Dashed lines indicate the contour of individual cells.
nt, nucleus tether (Nvj1$^{1\text{-}125}$).
b and **c** GFP-Atg8, GFP-Atg8 *ypt7Δ*, GFP-Atg8 *vac8Δ* and GFP-Atg8 *ypt7Δ vac8Δ* containing either nt-mScarlet-Vac8ΔN or nt-mScarlet were grown to mid log-phase. **b** Representative fluorescence microscopy images are shown. Dashed lines indicate the contour of individual cells. **c** The number of GFP-Atg8 puncta per cell was analysed in three independent biological replicates. For each condition and replicate at least 100 cells were analyzed. The values of each replicate (circle) and the mean (bars) were plotted. Statistical analysis using two-tailed unpaired t-tests. Significance is indicated with asterisks: *** $p < 0.001$, ** $p < 0.01$, * $p < 0.05$, n.s. (not significant) $p > 0.05$. Exact numerical values are reported in the source data.
