# Supplementary material for: Spatial control of avidity regulates initiation and progression of selective autophagy
Source: Nat Commun. 2021 Dec 10;12:7194. doi: 10.1038/s41467-021-27420-3 (PMC8664900; doi:10.1038/s41467-021-27420-3)
Supplement: Supplementary file 3 — Description of Additional Supplementary Files [file 41467_2021_27420_MOESM3_ESM.docx]

# Inventory of Supporting Information, Hollenstein et al.

**Supplementary Information File:**

Supplementary Figures 1-9

**Supplementary Data 1:**

Yeast strains used in this study

**Supplementary Data 2:**

Plasmids used in this study

**Supplementary Data 3:**

Plasmid sequence maps

**Source Data file:**

Uncropped Western blots.

Quantification and statistical tests
