# Supplementary material for: Spatial control of avidity regulates initiation and progression of selective autophagy
Source: Nat Commun. 2021 Dec 10;12:7194. doi: 10.1038/s41467-021-27420-3 (PMC8664900; doi:10.1038/s41467-021-27420-3)
Supplement: Supplementary file 7 — Reporting Summary [file 41467_2021_27420_MOESM7_ESM.pdf]

nature research

Corresponding author(s): Claudine Kraft

Last updated by author(s): Nov 5, 2021

Reporting Summary

Nature Research wishes to improve the reproducibility of the work that we publish. This form provides structure for consistency and transparency in reporting. For further information on Nature Research policies, see our Editorial Policies and the Editorial Policy Checklist.

Statistics

For all statistical analyses, confirm that the following items are present in the figure legend, table legend, main text, or Methods section.

n/a	Confirmed	
☐	☒	The exact sample size (*n*) for each experimental group/condition, given as a discrete number and unit of measurement
☐	☒	A statement on whether measurements were taken from distinct samples or whether the same sample was measured repeatedly
☐	☒	The statistical test(s) used AND whether they are one- or two-sided<br>*Only common tests should be described solely by name; describe more complex techniques in the Methods section.*
☒	☐	A description of all covariates tested
☒	☐	A description of any assumptions or corrections, such as tests of normality and adjustment for multiple comparisons
☐	☒	A full description of the statistical parameters including central tendency (e.g. means) or other basic estimates (e.g. regression coefficient) AND variation (e.g. standard deviation) or associated estimates of uncertainty (e.g. confidence intervals)
☐	☒	For null hypothesis testing, the test statistic (e.g. *F*, *t*, *r*) with confidence intervals, effect sizes, degrees of freedom and *P* value noted<br>*Give P values as exact values whenever suitable.*
☒	☐	For Bayesian analysis, information on the choice of priors and Markov chain Monte Carlo settings
☒	☐	For hierarchical and complex designs, identification of the appropriate level for tests and full reporting of outcomes
☒	☐	Estimates of effect sizes (e.g. Cohen's *d*, Pearson's *r*), indicating how they were calculated

Our web collection on statistics for biologists contains articles on many of the points above.

Software and code

Policy information about availability of computer code

Data collection	Fluorescence microscopy images were captured using the VisiView software (version 4.4.0.2, Visitron Systems), softWorX suite (version R6.1.1 or version 7.2.1). Raw microscopy images acquired with the DeltaVision Ultra High Resolution microscope or PersonalDeltaVision microscope were deconvolved using the softWorX deconvolution plugin.
Data analysis	All fluorescence image analysis was performed using FIJI (ImageJ version 1.53c).

For manuscripts utilizing custom algorithms or software that are central to the research but not yet described in published literature, software must be made available to editors and reviewers. We strongly encourage code deposition in a community repository (e.g. GitHub). See the Nature Research guidelines for submitting code & software for further information.

Data

Policy information about availability of data

All manuscripts must include a data availability statement. This statement should provide the following information, where applicable:

- Accession codes, unique identifiers, or web links for publicly available datasets
- A list of figures that have associated raw data
- A description of any restrictions on data availability

No restrictions apply to the data collected for this manuscript. Further raw data and resources from this study are available from the corresponding author on reasonable request. Raw data underlying the bar graphs and uncropped western blots are provided in the Source Data file.

Field-specific reporting

Please select the one below that is the best fit for your research. If you are not sure, read the appropriate sections before making your selection.

- [x] Life sciences
- [ ] Behavioural & social sciences
- [ ] Ecological, evolutionary & environmental sciences

For a reference copy of the document with all sections, see nature.com/documents/nr-reporting-summary-flat.pdf

Life sciences study design

All studies must disclose on these points even when the disclosure is negative.

Sample size	No statistical methods were used to predetermine the sample size. Instead sample size was based on the commonly applied standards in the field and our experience. In previous studies we have observed robust consistency between independent replicates of the assays used in this manuscript, hence we have established n=3 as sufficient to reveal differences between different mutants or conditions (Papinski et al., MolCell 2014; Torggler et al., MolCell 2016; Hollenstein et al., JCS 2019). For quantitative fluoresence microscopy analysis three independent biological replicates were analyzed, which allowed meaningful statistical comparisons. The electron microscopy experiment was performed to support the findings of the preceeding fluorescence microscopy experiments, for the quantitative electron microscopy analysis three independent technical replicates were analyzed. For western blot and qualitative fluoresence microscopy experiments two or more independent experiments were performed. Information about sample size is provided in the figure legends.
Data exclusions	All exclusion criteria were pre-established. Partially visible cells at the border of the microscopy imaging field were excluded from the analysis. For experiments, in which FM4-64 staining was performed, cells not containing vacuolar FM4-64 signal were excluded from the analysis because the FM4-64 staining served as a marker for vacuolar localization. For the nucleus tethering experiments, cells not containing nuclear mScarlet signal were excluded from the analysis. This exclusion criteria was established because first, the mScarlet signal served as a marker for nuclear localization and second, to exclude cells with very low or no expression of nucleus-tethered proteins.
Replication	Replication were successful. Presented western blotting data are representative for two or more experiments that produced the same result: All western blots were performed at least three times, except for the Ape1 blot in Fig. 7c, which was performed to verify the results obtained by fluorescence microscopy in Fig. 7b and performed twice with the same result. The presented yeast two hybrid data and the qualitative fluorescence microscopy data are representative for two or more experiments that produced the same result. For quantitative fluorescence microscopy and quantitative electron microscopy the quantification of each individual experiment is shown in the figures.
Randomization	Covariates were not considered to be relevant for this study due to the highly controllable and reproducible experimental conditions achieved by using S. cerevisiae as a model organism and the nature of the performed experiments, which is the standard in the field.
Blinding	For fluorescence microscopy, random fields of view were selected by brightfield microscopy before capturing fluorescence images. Manual quantification of fluorescence microscopy was performed blindly after randomizing image names. For yeast two hybrid and western blot experiments blinding was not carried out as the methods used to collect data were not subjective in nature, i.e. no further data analysis was performed and the raw data are shown in the figures.

Reporting for specific materials, systems and methods

We require information from authors about some types of materials, experimental systems and methods used in many studies. Here, indicate whether each material, system or method listed is relevant to your study. If you are not sure if a list item applies to your research, read the appropriate section before selecting a response.

Materials & experimental systems

n/a	Involved in the study	
☐	☒	Antibodies
☐	☒	Eukaryotic cell lines
☒	☐	Palaeontology and archaeology
☒	☐	Animals and other organisms
☒	☐	Human research participants
☒	☐	Clinical data
☒	☐	Dual use research of concern

Methods

n/a	Involved in the study	
☒	☐	ChIP-seq
☒	☐	Flow cytometry
☒	☐	MRI-based neuroimaging

Antibodies

Antibodies used	The following antibodies were used in this study:<br>mouse monoclonal anti-GFP (clone 2B6, Merck, cat # MABC1689)<br>mouse monoclonal anti-Pgk1 (clone 22C5D8, Invitrogen, cat # 459250)<br>rabbit polyclonal PAP (Sigma-Aldrich, cat # P1291)<br>rabbit polyclonal anti-Atg1 (Daniel Klionsky, University of Michigan, USA; first described in Abeliovich et al., MBoC 2003)<br>rabbit polyclonal anti-Tom20 (Chris Meisinger, University of Freiburg, Germany; first described in Voegtle et al, JCB 2015)

	rabbit polyclonal anti-Atg19 (Sascha Martens, University of Vienna, Austria)<br>rabbit polyclonal anti-Ape1 (Claudine Kraft, University of Freiburg, Germany; first described in Papinski et al., MolCell 2014)<br>mouse monoclonal anti-Atg11 (clone 6F4-G4, Claudine Kraft, University of Freiburg, Germany; first described in Torggler et al., MolCell 2016)<br>mouse monoclonal anti-Atg17 (clone 4D3-E8) and anti-Atg29 (clone 1C4-D5) were generated by the Kraft laboratory and described for the first time in this manuscript.
Validation	The anti-GFP antibody was tested and used for immunoblotting of HEK293T cells stably transfected with GFP-tagged Aquaporin 4 (https://www.merckmillipore.com/AT/de/product/Anti-GFP-Antibody-clone-2B6,MM_NF-MABC1689-25UG). We additionally validated the specificity of the anti-GFP antibody for yeast whole cell extracts with GFP-tagged Vac8, which generated a single band at the expected size in western blotting that was absent in control yeast lysates not expressing GFP (see Figure 2g).<br>The anti-PAP antibody was tested and used for immunoblotting of yeast whole cell extracts (https://www.sigmaaldrich.com/AT/de/product/sigma/p1291)<br>The anti-Pgk1 antibody was tested and used for immunoblotting of yeast whole cell extracts (https://www.thermofisher.com/antibody/product/PGK1-Antibody-clone-22C5D8-Monoclonal/459250)<br>The anti-Ape1 antibody was generated in the Kraft laboratory. It has been used in various publication from our lab and has been validated previously for western blotting of yeast whole cell extracts (Torggler et al., MolCell 2016).<br>The anti-Atg11 antibody was generated in the Kraft laboratory. It has been validated previously for western blotting of yeast whole cell extracts (Torggler et al., MolCell 2016).<br>The anti-Atg19 antibody was generated in the Martens laboratory. We have validated it for western blotting in yeast, which produced a signal at the expected molecular size of the target protein that was absent in the respective knock-out strain.<br>The anti-Tom20 antibody was generated in the Meisinger laboratory. It has been validated previously for western blotting of yeast whole cell extracts (Voegtle et al., JCB 2015 and NatCom 2017; Poveda-Huertes et al., MolCell 2019).<br>The anti-Atg1 antibody was generated in the Klionsky laboratory. It has been validated previously for western blotting of yeast whole cell extracts (Cheong et al., MBoC 2005).<br>The anti-Atg17 and anti-Atg29 antibodies generated in the Kraft laboratory and first described in this manuscript. They have been validated for western blotting in yeast, which produced a signal at the expected molecular size of the target protein that was absent in the respective knock-out strain.

Eukaryotic cell lines

Policy information about cell lines

Cell line source(s)	Sf9 Spodoptera frugiperda insect cells were obtained from Expression Systems.
Authentication	None of the cell lines were authenticated
Mycoplasma contamination	None of the cell lines were tested for mycoplasma contamination
Commonly misidentified lines<br>(See ICLAC register)	None of the cell lines used in this study belong to the group of commonly misidentified lines
