# Supplementary material for: Spatial control of avidity regulates initiation and progression of selective autophagy
Source: Nat Commun. 2021 Dec 10;12:7194. doi: 10.1038/s41467-021-27420-3 (PMC8664900; doi:10.1038/s41467-021-27420-3)
Supplement: Supplementary file 8 — Source Data [file 41467_2021_27420_MOESM8_ESM.zip › Source data/Uncropped_western_blots.pdf]

# Uncropped western blots

## Fig. 2g

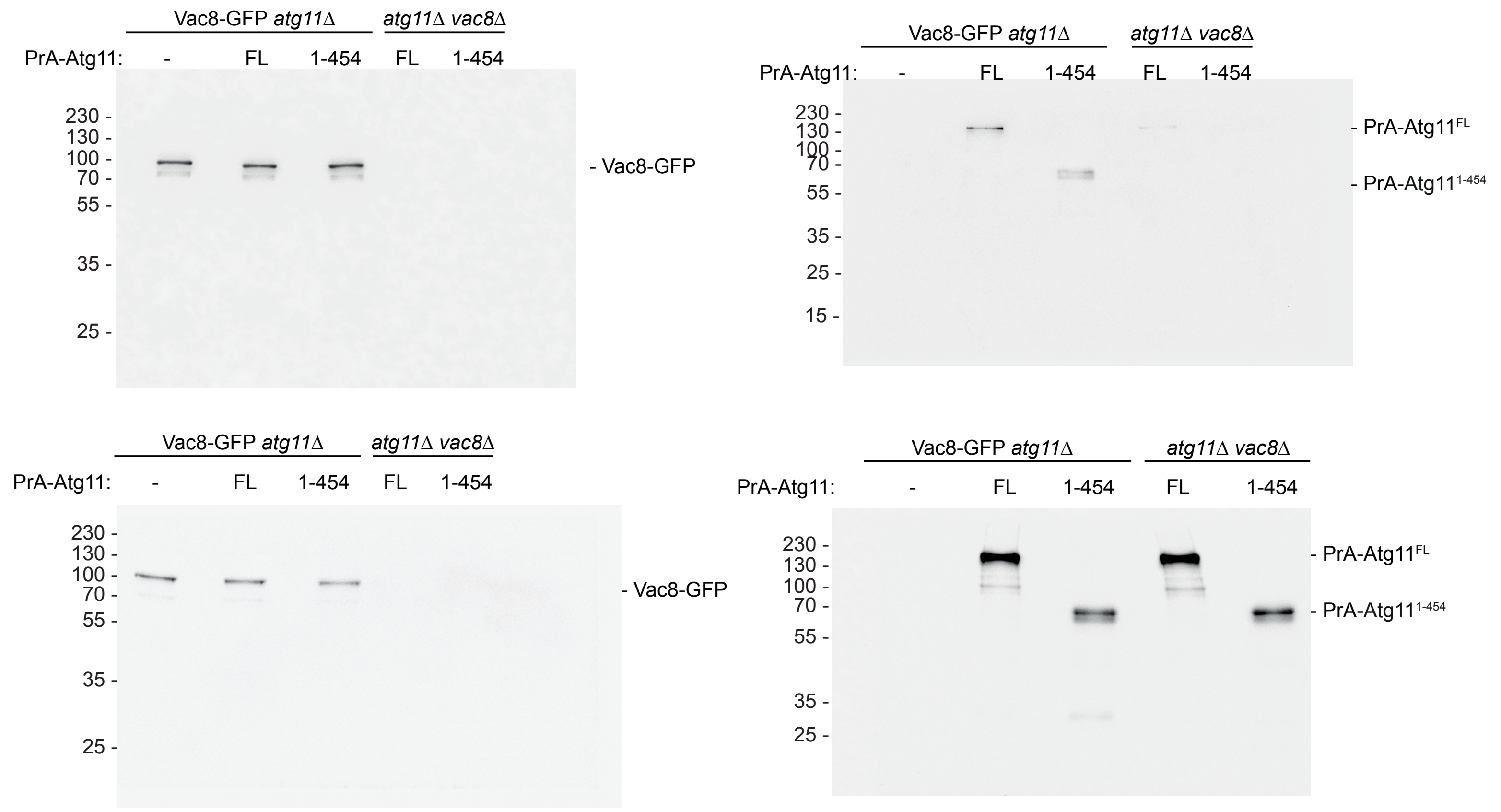


## Supplementary Fig. 3c

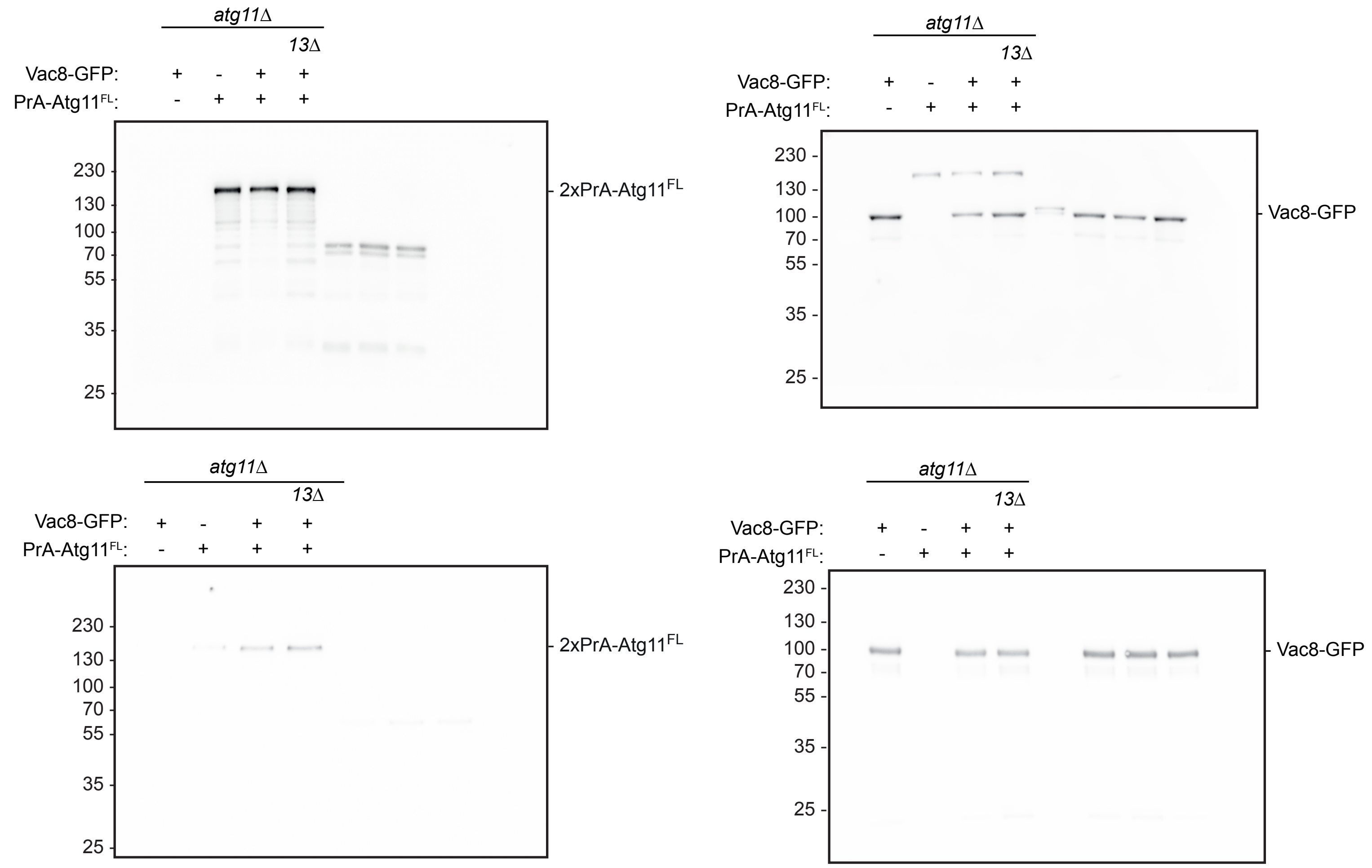

## Supplementary Fig. 3d

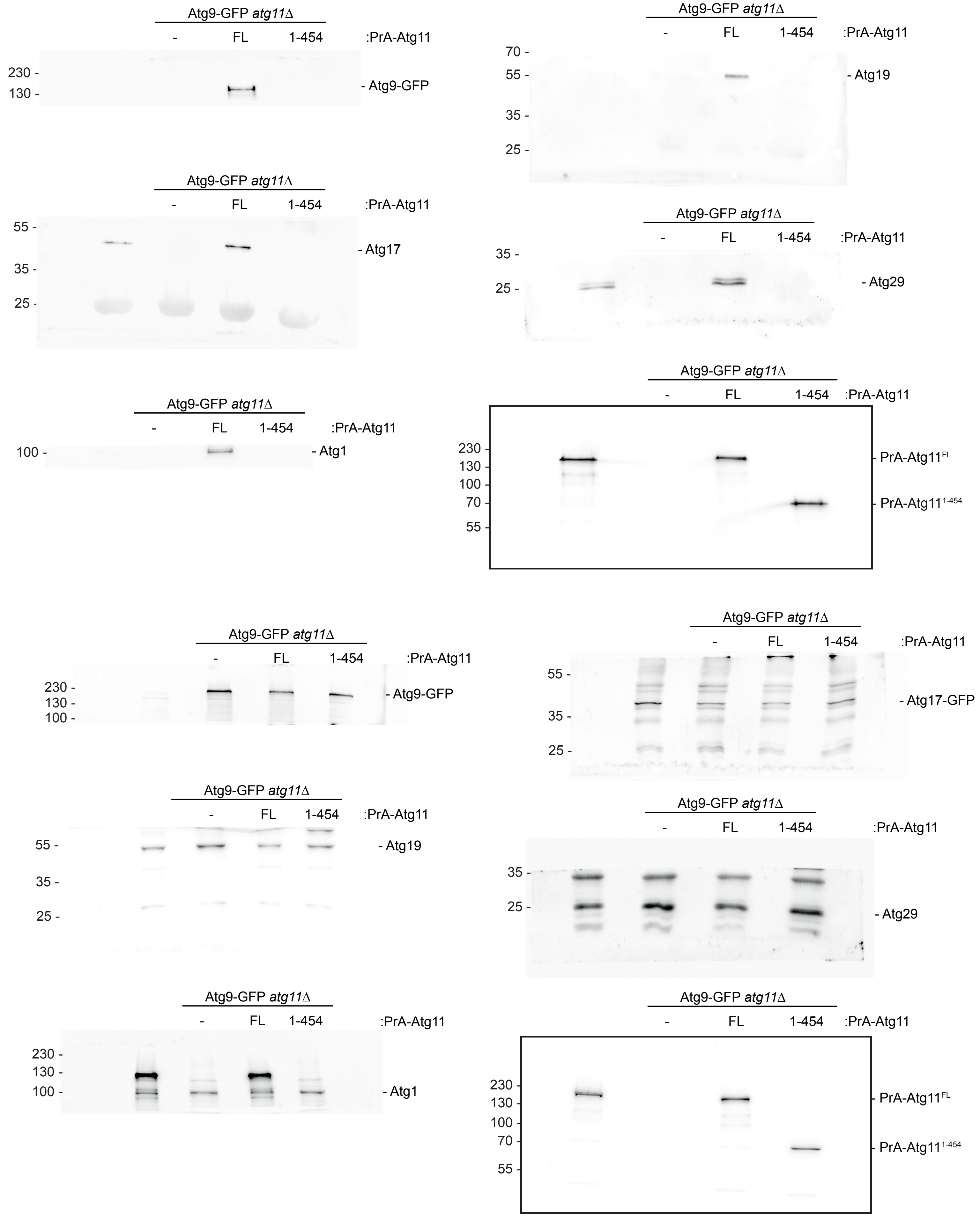

## Fig. 3a

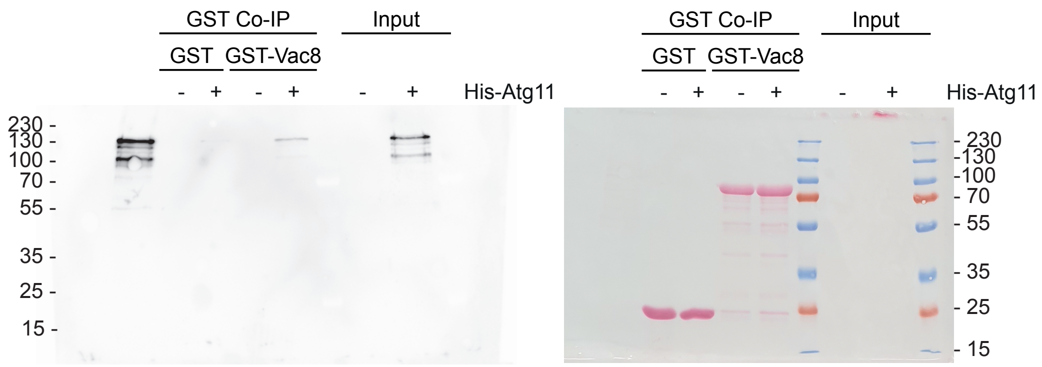
GST Co-IP
Input
GST
GST-Vac8
- + - + - +
His-Atg11
230 -
130 -
100 -
70 -
55 -
35 -
25 -
15 -

## Fig. 3b

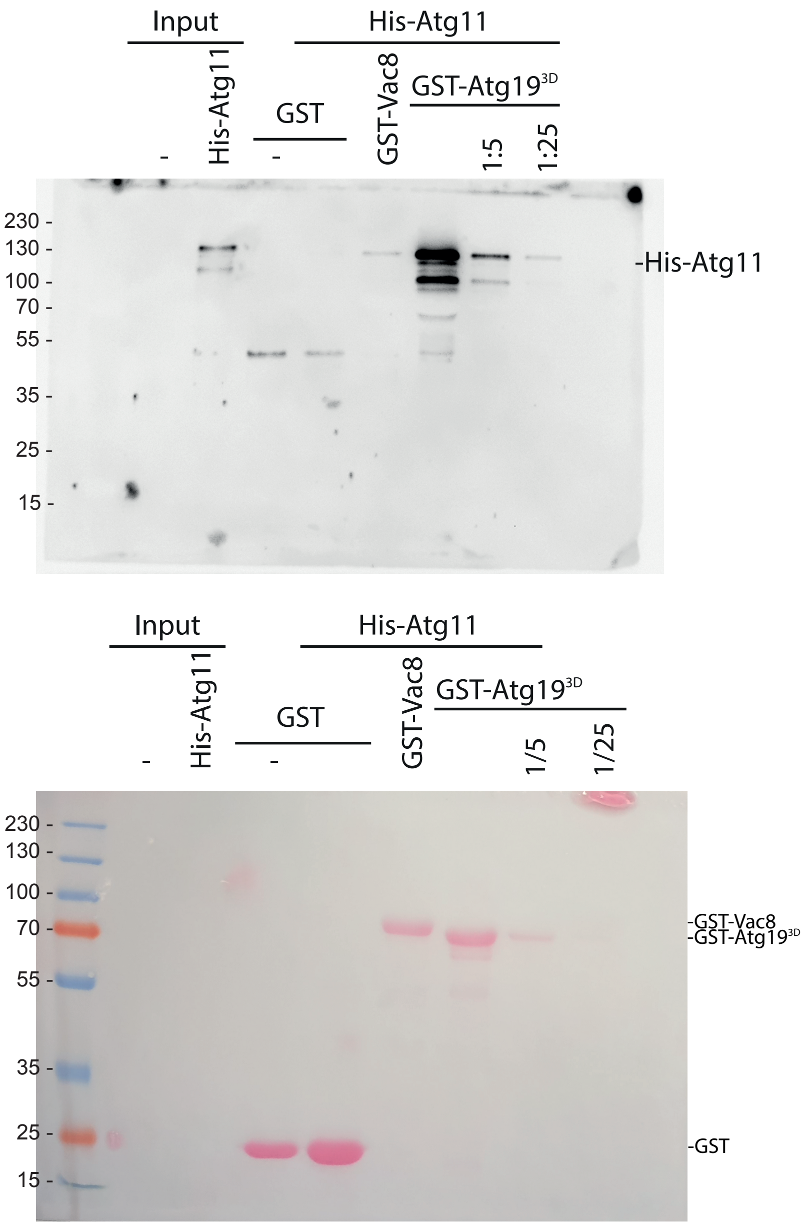
Input
His-Atg11
-
His-Atg11
GST
-
GST-Vac8
GST-Atg19$^{3D}$
1:5
1:25
230 -
130 -
100 -
70 -
55 -
35 -
25 -
15 -
-His-Atg11
1/5
1/25
-GST-Vac8
-GST-Atg19$^{3D}$
-GST

## Fig. 4f

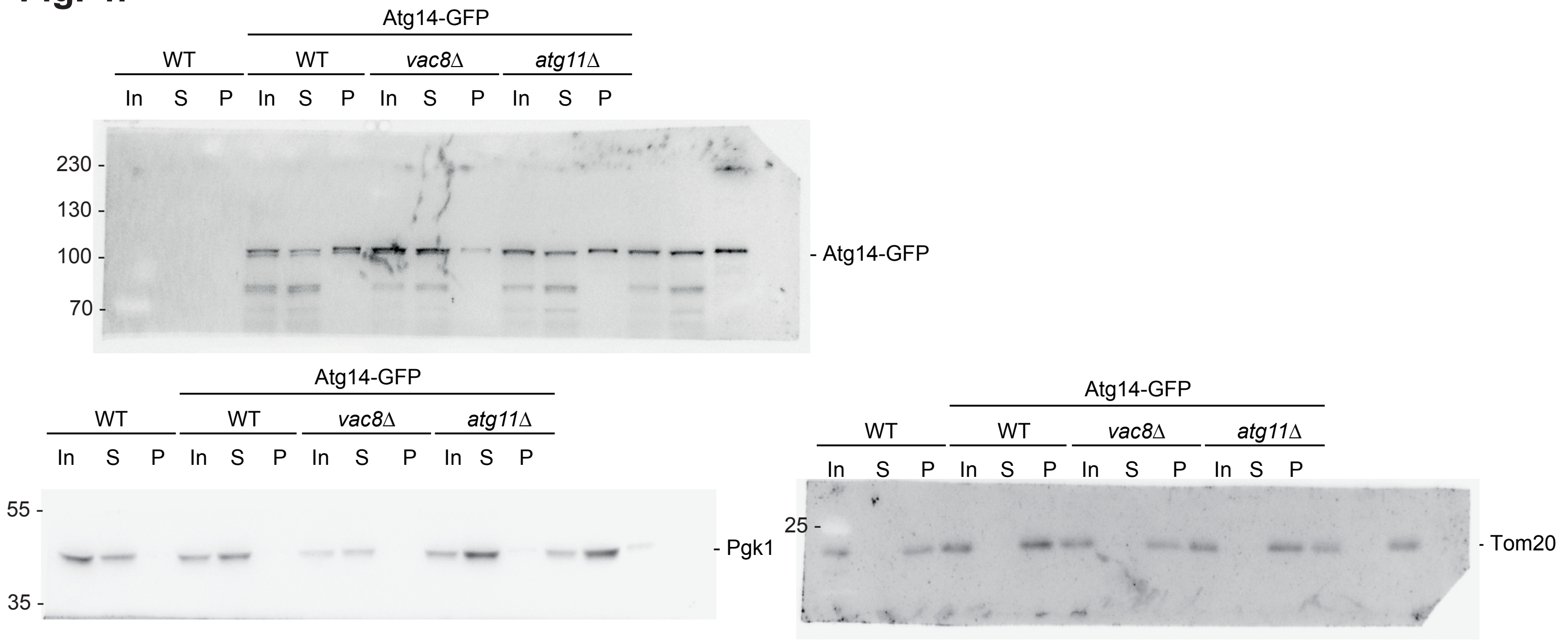

Atg14-GFP
WT
WT
vac8Δ
atg11Δ
In S P In S P In S P In S P
230 -
130 -
100 -
70 -
- Atg14-GFP
55 -
35 -
- Pgk1
25 -
- Tom20


## Fig. 4h

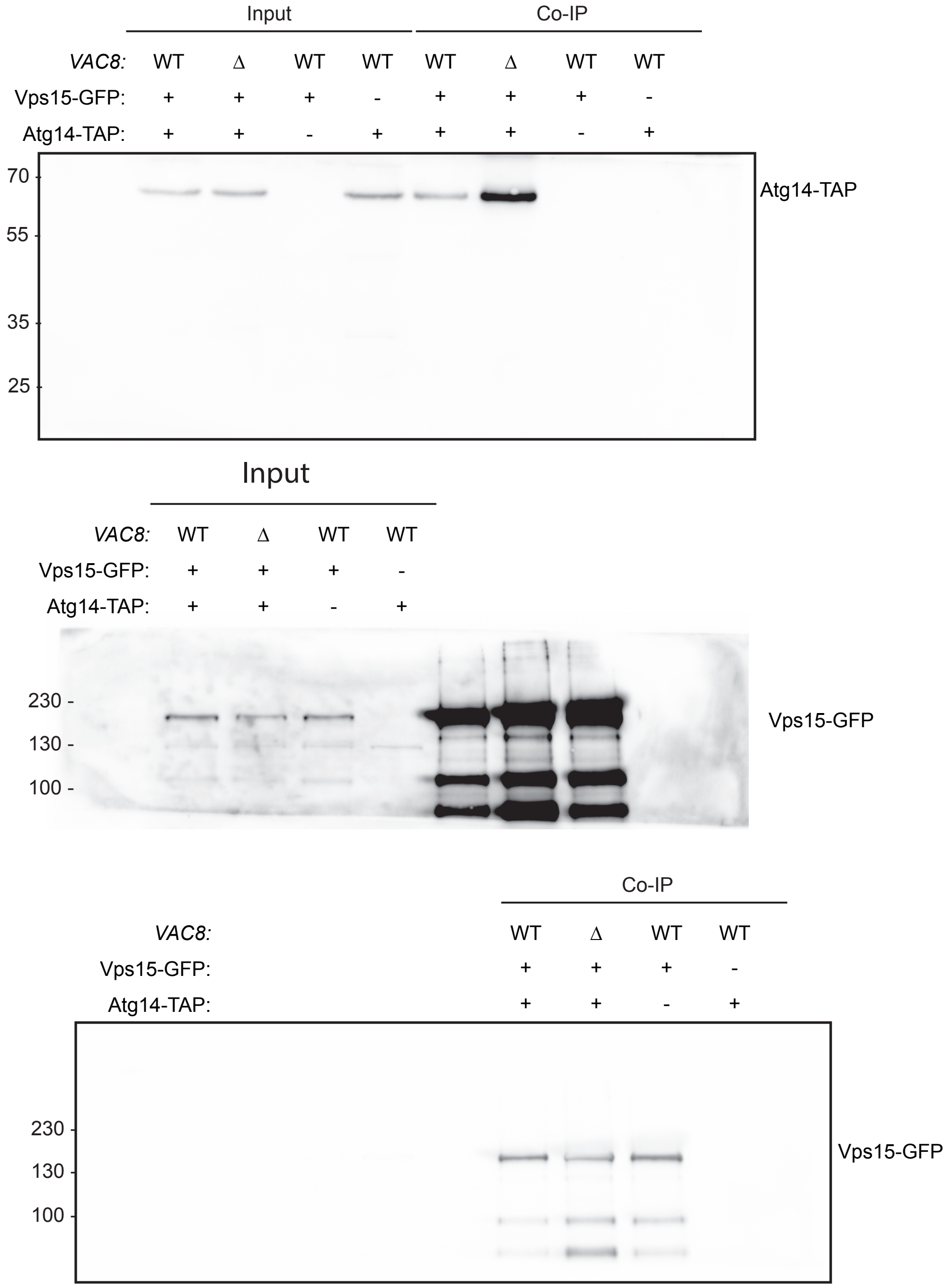

Input
Co-IP
VAC8: WT Δ WT WT WT Δ WT WT
Vps15-GFP: + + + - + + + -
Atg14-TAP: + + - + + + - +
70
55
35
25
Atg14-TAP
Input
VAC8: WT Δ WT WT
Vps15-GFP: + + + -
Atg14-TAP: + + - +
230 -
130 -
100 -
Vps15-GFP
Co-IP
VAC8: WT Δ WT WT
Vps15-GFP: + + + -
Atg14-TAP: + + - +
230
130
100
Vps15-GFP

## Fig. 4h

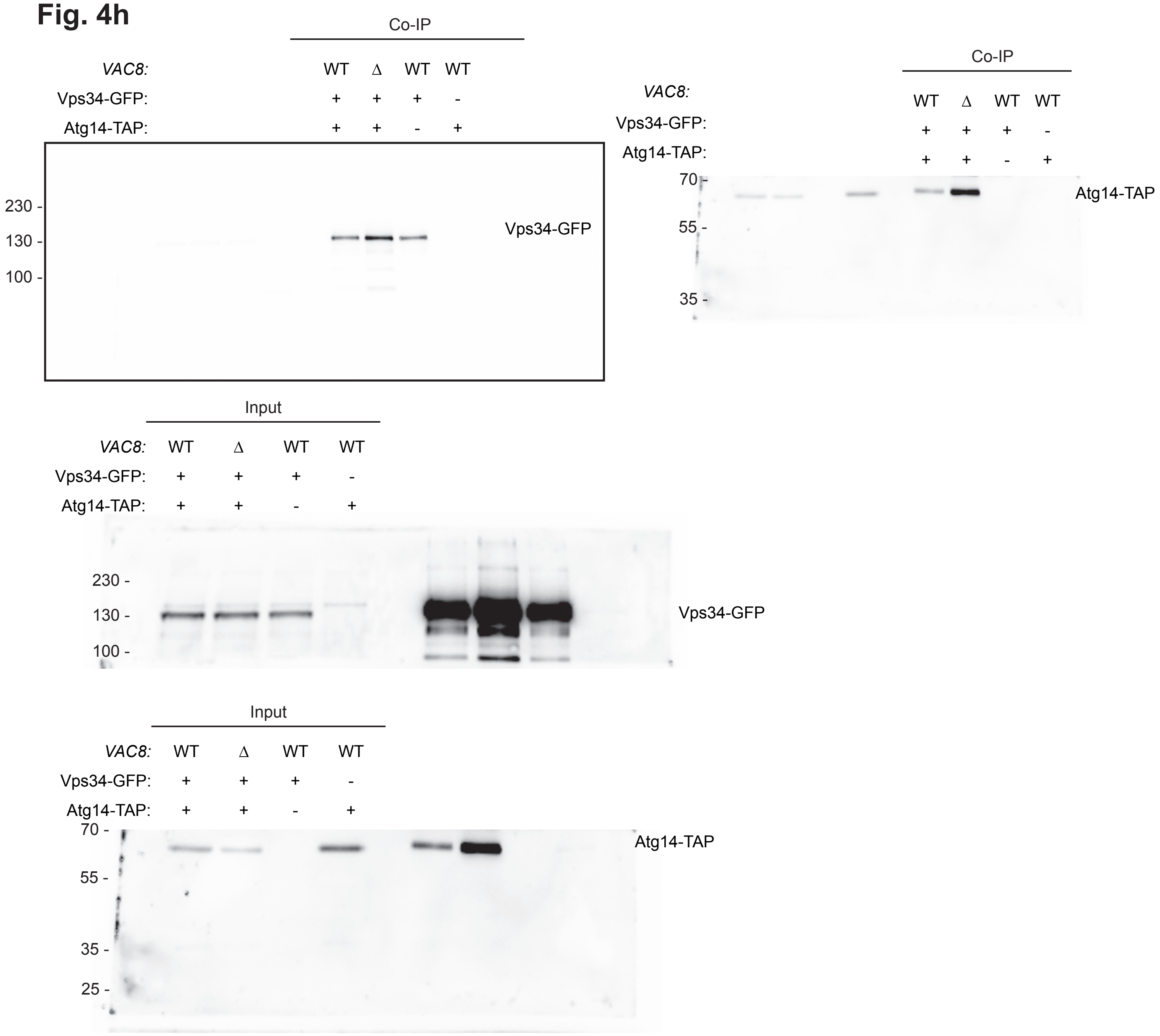
Co-IP
VAC8: WT Δ WT WT
Vps34-GFP: + + + -
Atg14-TAP: + + - +
230 -
130 -
100 -
Vps34-GFP
Co-IP
VAC8: WT Δ WT WT
Vps34-GFP: + + + -
Atg14-TAP: + + - +
70 -
55 -
35 -
Atg14-TAP
Input
VAC8: WT Δ WT WT
Vps34-GFP: + + + -
Atg14-TAP: + + - +
230 -
130 -
100 -
Vps34-GFP
Input
VAC8: WT Δ WT WT
Vps34-GFP: + + + -
Atg14-TAP: + + - +
70 -
55 -
35 -
25 -
Atg14-TAP

## Fig. 7c

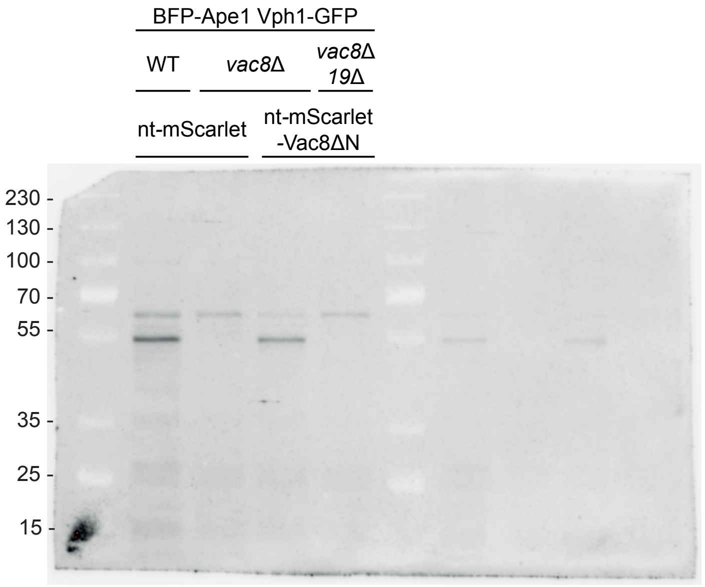
BFP-Ape1 Vph1-GFP
WT
vac8Δ
vac8Δ 19Δ
nt-mScarlet
nt-mScarlet -Vac8ΔN
230 -
130 -
100 -
70 -
55 -
35 -
25 -
15 -
